# Early Infant Diagnosis Sample Management in Mashonaland West Province, Zimbabwe, 2017

**DOI:** 10.1155/2018/4234256

**Published:** 2018-07-26

**Authors:** Hamufare Mugauri, Owen Mugurungi, Addmore Chadambuka, Tsitsi Juru, Notion Tafara Gombe, Gerald Shambira, Mufuta Tshimanga

**Affiliations:** ^1^Department of Community Medicine, University of Zimbabwe, Zimbabwe; ^2^Ministry of Health and Child Care, Zimbabwe; ^3^Elizabeth Glaser Paediatric AIDS Foundation (EGPAF), Zimbabwe

## Abstract

**Background:**

In 2016, Mashonaland West Province had 7.4% (520) dried blood spot (DBS) samples for early infant diagnosis (EID) rejected by the Zimbabwe National Microbiology Reference Laboratory (NMRL). The samples were suboptimal, delaying treatment initiation for HIV-infected children. EID is the entry point to HIV treatment services in exposed infants. We determined reasons for DBS sample rejections and suggested solutions.

**Methods:**

A cause-effect analysis, modelled on Ishikawa, was used to identify factors impacting DBS sample quality. Interviewer-administered questionnaires and evaluation of sample collection process, using Standard Operating Procedure (SOP) was conducted. Rejected samples were reviewed. Epi Info™ was used to analyze findings.

**Results:**

Eleven (73.3%) facilities did not adhere to SOP and (86.7%) did not evaluate DBS sample quality before sending for testing. Delayed feedback (up to 4 weeks) from NMRL extended EID delay for 14 (93.3%) of the facilities. Of the 53 participants, 62% knew valid sample identification. Insufficient samples resulted in most rejections (77.9%). Lack of training (94.3%) and ineffective supervision (69.8%) were also cited.

**Conclusion:**

Sample rejections could have been averted through SOP adherence. Ineffective supervision, exacerbated by delayed communication of rejections, extended EID delay, disadvantaging potential ART beneficiaries. Following this study, enhanced quality control through perstage evaluations was recommended to enhance DBS sample quality.

## 1. Introduction

In 2015 alone, approximately 1.4 million mothers living with HIV gave birth and 150 000 infants became infected with HIV globally [1]. Unless these children are promptly commenced on lifelong antiretroviral treatment (ART), HIV-positive infants invariably record their highest mortality in the first three months of life. However, a serious diagnostics gap exists [1]. Only 51% of infants exposed to HIV globally are tested by the time they are six weeks old, the age recommended by the World Health Organization (WHO) [2]. Half of these never receive their results. Of those who test positive and receive their results, only half are linked to care [2].

Affordable ART and treatment of opportunistic infections are becoming increasingly available but this only benefits infants who are diagnosed early. In order to achieve the fast-track targets of ending the AIDS epidemic by 2030, new HIV infections among children must be eliminated. HIV can be transmitted from mother to child during pregnancy, childbirth, and breastfeeding, yet, with antiretroviral therapy, mother-to-child transmission rates can decrease, in likelihood, to 5% or less [3].

HIV infection can be definitively diagnosed through the use of virologic assays in most nonbreastfed HIV-exposed infants at 1 to 2 months of age and in virtually all infected infants by 4 months of age. HIV antibody tests, including newer tests, do not establish the presence of HIV infection in infants because of transplacental transfer of maternal antibodies to HIV; therefore, a virologic test should be used [4]. The samples need to be of optimal quality to ensure an accurate diagnosis. Positive virologic tests (i.e., nucleic acid tests (NAT))—a class of tests that include HIV Ribonucleic Acid (RNA) and Deoxyribonucleic Acid (DNA) polymerase chain reaction [PCR] assays, and related RNA qualitative or quantitative assays detect the HIV virus [4].

Early diagnosis of HIV allows health care providers to offer optimal care and treatment of HIV-infected children, assists in decision-making on infant feeding, and reduces illness-related stress in mothers and families. The increasing efficacy and coverage of Prevention of Mother-to-Child Transmission of HIV (PMTCT) interventions have resulted in the majority of children born to HIV-infected mothers being HIV-free [5]. Consequently, identification of infected children before the illness is possible through routine diagnostic testing of all exposed infants. WHO recommends that this be performed with virological testing at 6 weeks of age and HIV-positive infants are commenced on lifelong ART, as recommended by WHO, on provider-initiated HIV testing [6].

Among the technologies available for diagnosis of HIV in infants, PCR on DNA in blood is the most widely used and is generally considered to be the standard method. Recent studies have also demonstrated evidence that real-time PCR for HIV RNA provides a reliable and suitable alternative. HIV DNA-PCR is a qualitative test (i.e., it gives a yes/no diagnosis of HIV infection) [7]. HIV RNA detection provides additional quantitative information on virological status, and the same technology/equipment is used for monitoring the response to treatment and possible therapeutic failure [8].

Both DNA and RNA technologies are complex and expensive, requiring dedicated equipment, space, and trained technicians. However, increased automation greatly reduces the technical challenge. Some DNA and RNA (PCR) technologies support the use of DBS samples, which have considerable advantages in settings where sample-taking, transportation, and storage are problematic [9].

In Zimbabwe, ART services are increasingly being offered at primary health care facilities. Because of rapid disease progression in infants, early identification of infected infants is recognized [10]. In 2007, Zimbabwe introduced virological testing which detects a component of the HIV deoxyribonucleic acid (DNA) through the use of a polymerase chain reaction (DNA-PCR) from 6 weeks of age in HIV-exposed infants [11]. The tests are conducted at the National Microbiology Reference Laboratory (NMRL) in Harare and of late at Mpilo Central Hospital and Mutare Provincial Hospital [12].

Samples for the DNA-PCR are collected from the infants in the form of a dried blood spot (DBS) which is then sent to the National Microbiology Reference Laboratory (NMRL) via district or provincial laboratories. The sample is accompanied by a laboratory request form which captures patient identification variables (request number, province, district, health facility, mother's name, infant's name, sex, and date of birth). It also captures the date the sample was taken, mode of delivery, PMTCT prophylaxis, and infant's feeding practice, reasons for the DNA-PCR test, entry point for the test, and finally the result of the test [12].

**Figure 1:**
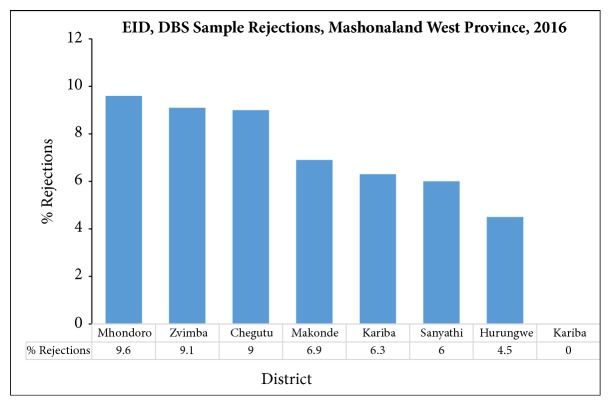
DBS rejections per district, Mashonaland West Province, Zimbabwe, 2016.

A decline in the quality of dried blood spot (DBS) specimens for early infant diagnosis (EID) of HIV was observed in 2016. A total of 2020 (3.5%) samples were rejected by the National Microbiology Reference Laboratory (NMRL). Mashonaland West Province recorded the highest rejections countrywide at 520 (7.35%) surpassing the national rejections proportion twofold. Discrepancies in per-districts rejections were also enumerated, as shown in Figure 1.

The rejections were for varied reasons, chiefly insufficient samples, contamination, and mismatch between sample and form, which indicate divergence from minimal quality standards as stipulated by the Standard Operating Procedure (SOP) for collection, storage, and transportation of DBS samples [13].

Rejection of samples leads to delay in providing critical lifelong antiretroviral therapy to deserving children and is contributory to the prevailing 43% paediatric ART initiations, against 74% for adults in Zimbabwe [12]. The rise in the rejection rates, above the World Health Organization (WHO) maximum threshold of 2%, presents an urgent need to evaluate the existing EID sample management mechanisms and the extent of adherence to them in order to enhance paediatric ART initiations [13]. It is within this background that we sort to analyze the processes of collection and transportation of DBS specimens for EID of HIV using the SOP as a guide, in order to inform recommendations for DBS sample quality enhancement.

## 2. Material and Methods

**Figure 2:**
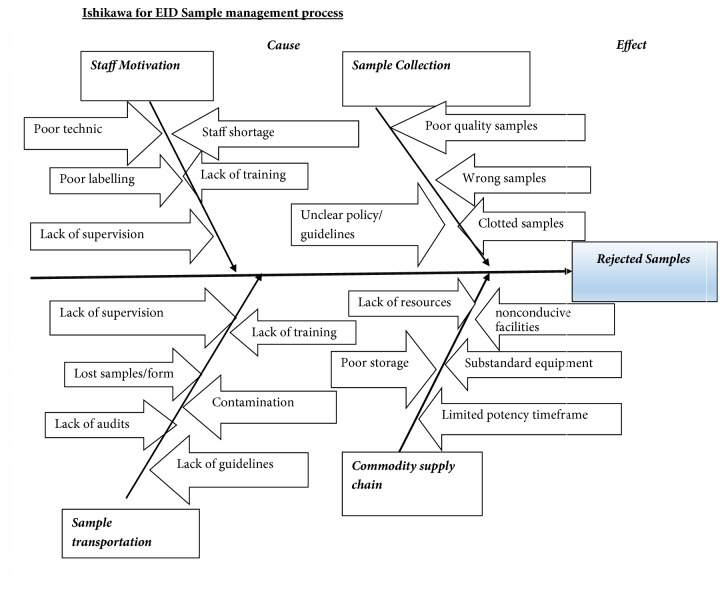
Ishikawa for EID sample management, Mashonaland West Province, Zimbabwe, 2017.

A cause-effect analysis, using an Ishikawa diagram was conducted to evaluate factors contributing to poor quality DBS samples (Figure 2.)

This study was conducted in Mhondoro, Zvimba, and Chegutu, the leading districts on DBS rejections in Mashonaland West Province.

A total of 15 health facilities offering EID services in the three districts were randomly selected and included in the study which was conducted between October and December 2017.

The sample size was calculated using the Dobson formula: n = Za2 (p) (1-p)/delta2, where Za=1.96, p=0.5, assuming that 50% of health workers to be interviewed have adequate knowledge, at 20% precision and 80% power, a sample size of 47, adjusted for 10% nonresponse rate, and sample size of 52 was reached. A total of 53 participants were included in the study, yielding 102% response rate. Registered General Nurses (Diploma), Primary Care Nurses (PCN), and Primary Counsellors (PC) at all health facilities offering EID were recruited into the study. The District Medical officers (DMO), District Nursing Officers (DNO) from the district hospitals, and health facility Sisters in Charge (SIC) were recruited as key informants.

Timely feedback, from NMRL to sample collecting facilities, was defined as the reception of a sample acceptance or rejection decision within 10 days of submission of a sample. The definition is consistent with recommendations of the Standard Operating Procedure (SOP).

A pretested interviewer-administered questionnaire was used to collect data from healthcare workers (nurses and Primary counsellors) and key informant interviews were conducted with nurse managers. The pretest was done at three health facilities within the districts, which were then excluded from the study, and adjustments to the questionnaire were made, some questions were edited for clarity. Epi Info was used to generate frequencies, means, and proportions. DBS sample rejection results were reviewed and checklists utilized to evaluate SOP adherence. Permission to carry out the study was obtained from the Ministry of Health and Child Care, and Institutional review board of Mashonaland West Province. Written informed consent was obtained from key informants.

## 3. Results and Discussion

### 3.1. Demographic Characteristics

The 53 successfully recruited participants were evenly distributed across the three districts. Overall, 66% (35) were females whilst 34% (18) were males. The majority, 54.7% (29) of participants were Primary care nurses and Primary counsellors were the least at 13.2% (7). A District Nursing Officer (DNO) and District Medical Officer (DMO) from each district were recruited as key informants (Table 1).

### 3.2. Compliance with Standard Operating Procedure (SOP) for DBS Sample Collection

In all facilities visited, nurses allocated to the Family and Child Health (FCH) unit at health facilities were charged with the responsibility of collecting DBS samples regardless of prior training on the requisite technic. Only trained (formally/informally) personnel are supposed to collect DBS samples, following the Standard Operating Procedures (SOP).

Only two (13.3%) facilities observed the requirement that a second nurse or charge nurse verify the collected samples for quality and the documentation for completeness and consistency.

Facility-level documentation of collected samples was practiced at four (26.7%) of the facilities. A local register of collected samples and their results is the recommended practice. One (6.7%) facility received timely feedback from the laboratory. The rest of the facilities only obtained results when the entire batch results were received back at the facility (Table 2).

### 3.3. Level of Expertise of Personnel Responsible for the Collection of DBS Samples for EID

The level of expertise of personnel responsible for DBS collection was assessed through the checklist, interview, and observation. The majority (41; 77.4%) of the participants accurately enumerated the DBS collection process in sequence whilst 75.5% (40) were able to identify key characteristics of a potent DBS sample. Precautionary measures necessary to obtain a valid DBS sample were identified by 56.6% (30) of the participants. Thirty-two (60.4%) of the participants accurately related the packaging process of DBS samples. Notably, only 37.7% (20) of the participants perceived the entire sequential DBS process as necessary to obtain valid specimens (Table 3).

### 3.4. Reasons for DBS Sample Rejections at NMRL

A total of 27.7% (145) Mashonaland West Province DBS rejections were shared among the 3 districts. The most prevalent reason for rejection was insufficient samples at 77.9% (113). Nearly 10% (14) of the samples were rejected because the specimen had clotted, whilst mismatching form and specimen constituted 6.9% of the rejections. Cross-contamination constituted 2.1% (3) of the rejections, and failure to submit the requisite specimen constituted less than 1% (1) of the rejections. All rejection reasons indicated divergence from the Standard Operating Procedures (SOP) for DBS sample collection (Table 4).

### 3.5. Reasons for Failing to Collect Potent DBS Samples

Ninety-four percent (50) of the participants stated that lack of training on DBS collection was the reason for the high rejection rates, whilst almost 70% (37) thought that lack of supervision resulted in errors, leading to the high rejection rates. Failing to execute the correct technic during collection process was cited by 67.9% (36) of the participants. The poor technic was implicated for failure to obtain an adequate quantity of the DBS, resulting in insufficient samples. The perceived complexity of the DBS collection process and unclear rejection criteria by the laboratory were cited by 49.1% and 50.9% of the participants, respectively.

## 4. Discussion

The study sought to determine factors associated with increased DBS sample rejections in Mashonaland West Province using an Ishikawa for cause-effect analysis.

The DBS collection process is supposed to be executed sequentially, observing the SOP prescribed precautionary measures in order to obtain valid samples. This study observed divergence from the prescribed criteria, as seen by nontrained health workers being charged with the responsibility to collect the specimen. This anomaly, coupled with the absence of effective supervision to verify what the collecting nurse would have done before sending to the laboratory, resulted in high rejections. This finding concurs with the results by Grüner et al. (2015) in a systematic review of six studies who found a correlation between sequentially following DBS collection protocol and obtaining potent samples and authentic results [13].

When received DBS specimens did not meet the testing criteria during the screening process at NMRL, feedback to collecting site was not being timely conveyed to facilitate recollection. Health facilities only learnt of the rejection of reception of the entire batch or results. This resulted in a delay in the critical diagnosis of the HIV-exposed infant, impacting negatively on the EID programme.

DBS samples were rejected for preventable reasons, which required adherence to the SOP. Insufficient sample quantity was the single most common reason for rejections despite pictorial illustrations of the required sample amount in the SOP. This could have been averted by adhering strictly to the preparatory protocol, designed to ensure that an adequate amount of blood is obtained. This finding was contrary to the results of a study by Inalegwu (2016) in Nigeria who found insufficient samples as the least rejection reason, ahead of improper sample collection (26.3%) and improper labelling (16.4%) [14].

The reasons resulting in sample rejections, as perceived by the participants indicated an underlying competence deficiency. This revealed weaknesses in the currently practicced in-service skills dissemination, from nurses trained on DBS sample collection process, to their untrained counterparts, resulting in suboptimal samples being collected. This finding was consistent with findings from a systematic review by Smit et al. (2014), who found a strong correlation between adequate process orientation and high-quality samples [15]. There is, therefore, an urgent need to train all health workers involved in the DBS collection process.

## 5. Conclusions

DBS samples were being rejected for preventable reasons that reflected nonadherence to the minimum requirements of the SOP for sample collection, drying, storage, packaging, and transportation. The absence of a designated supervisory cadre to evaluate the quality of DBS samples before transportation to NMRL resulted in suboptimal specimens being transported to the laboratory, only to be rejected. The rejection decision was not timely conveyed to the collecting facility, resulting in delayed early infant diagnosis.

As a public health action, we discussed the supervision deficiencies with District Nursing Officers for the three districts, imploring them to strengthen quality control measures in place and ensuring even distribution of trained nurses across all health facilities. Feedback was also given to the districts and to the province. Enhanced quality control measures through per-stage evaluations were recommended to ensure valid DBS samples. From the collecting sites, all DBS samples were recommended to pass through the District Laboratory Scientists for quality check before conveying them to NMRL. Suboptimal samples are timely returned to collecting facilities for recollection. Upon arrival at NMRL, DBS samples are immediately graded and substandard ones are rejected and the feedback communicated immediately to collecting facilities for recollection.

## Figures and Tables

**Table 1:** Demographic Characteristics of participants, Mhondoro, Zvimba, and Chegutu districts, Mashonaland West Province, 2017.

**Variable**	**Category**	**Mhondoro** **n=19 **	**Zvimba** **n=17**	**Chegutu** **n=17**	**Total (**%**)** **n=53**
**Sex**	Male	6	7	5	18 (34)
	Female	13	10	12	35 (66)

**Job Position**	District Medical Officer	1	1	1	3 (5.7)
	District Nursing Officer	1	1	1	3 (5.7)
	Sister in Charge	2	1	3	17 (32.1)
	Registered General Nurse	4	2	4	10 (18.9)
	Primary Care Nurse	8	10	6	29 (54.7)
	Primary Counsellor	3	2	2	7 (13.2)

**Training **	Personnel trained	5	7	6	18(34)

**Median years in service**		6 (Q_1_=5;Q_3_=9)	5 (Q_1_=4;Q_3_=10)	9 (Q_1_=6;Q_3_=9)	7 (Q_1_=3;Q_3_=11)

**Table 2:** Compliance with Standard Operating Procedure (SOP) for DBS Sample Collection by Mhondoro, Zvimba and Chegutu Districts, 2016.

**Standard Operating Practice**	**Number of Compliant Facilities**	**Percentage Compliance** **n=15**
Only trained health care workers should collect DBS samples	0	0

The Charge nurse should verify the quality and completeness of collected samples before sending to the laboratory	2	13.3

A local register of collected samples and the received results should be kept	4	26.7

NMRL personnel to provide timely feedback for sub-quality samples after receiving them	1	6.7

**Table 3:** Expertise of Personnel Responsible for Collection of DBS samples for EID for Mhondoro, Zvimba, and Chegutu Districts, 2016.

**Variable**	**Mhondoro** **n=19 **	**Zvimba** **n=17**	**Chegutu** **n=17**	**Total (**%**)** **n=53**
**Accurately relates DBS collection sequence**	15	12	14	41 (77.4)

**Able to identify a valid DBS sample**	10	14	16	40 (75.5)

**Identify precautionary measures necessary for valid samples**	10	9	11	30 (56.6)

**Accurately relates sample packaging process**	12	10	10	32 (60.4)

**Perceive sequential DBS process essential for sample validity**	5	8	7	20 (37.7)

**Table 4:** Reasons for DBS samples rejections, reported by NMRL for Mhondoro, Zvimba, and Chegutu Districts, 2016.

**Reason for Rejection**	**Mhondoro** **N=50**	**Zvimba** **N=48**	**Chegutu** **N=47**	**Total (**%**)** **N=145**
**Insufficient sample**	38	35	40	113 (77.9)

**Clotted Blood**	7	4	3	14 (9.7)

**Mismatching form and specimen**	2	4	4	10 (6.9)

**Request form not submitted**	1	3	0	4 (2.8)

**Cross-contamination**	2	1	0	3 (2.1)

**Specimen not submitted**	0	1	0	1 (0.7)

## Data Availability

The data used to support the findings of this study are available from the corresponding author upon request.

## Abbreviations

ART: Antiretroviral therapy
ARV: Antiretroviral (drugs)
DBS: Dried blood spot
DNA-PCR: Deoxyribonucleic Acid Polymerase Chain Reaction
EID: Early infant diagnosis
NMRL: National Microbiology Reference Laboratory.
